# The In Vitro Adaptation of Patient-Derived Organoids Suggests Alternative Strategies against CMS1 Colorectal Cancer: When the Microenvironment Does Make the Difference

**DOI:** 10.3390/cancers14246086

**Published:** 2022-12-10

**Authors:** Alessandro Poggi, Serena Matis, Roberto Benelli

**Affiliations:** SSD Oncologia Molecolare e Angiogenesi, IRCCS Ospedale Policlinico San Martino, 16132 Genova, Italy

## 1. Introduction

Colorectal cancer (CRC) is a relatively slow-growing tumor that can be treated successfully when identified in the early stages. Unfortunately, its frequent localization in the right colon allows CRC to grow without detectable symptoms for years, retarding diagnosis. CRC was clustered into four consensus molecular subtypes (CMSs) by a large consortium of experts, who merged different classifiers derived by gene arrays: CMS1 (MSI-immune), CMS2 (canonical), CMS3 (metabolic) and CMS4 (mesenchymal). These classifiers are associated with clinical outcomes. As compared to other locations, the right colon is enriched in two particular CRC subtypes: CMS1 and CMS3 [1]. Due to the relatively oxygen-rich niche of the right colon, where aerobic bacteria are prevalent, CMS3 tumors enrichment is expected to occur. The frequent appearance of CMS1 tumors, which are very rare in other locations, is less explainable.

Most CMS1 tumors are characterized by microsatellite instability (MSI), BRAF^V600E^ mutation and/or mucinous differentiation. The presence of one or more of these pathological markers confers on the tumor a different aggressiveness and therapeutic response [2,3]. A fraction of MSI CRCs with a high mutation burden (MSI-high, about 5% of all CRC) show a more favorable prognosis due to the appearance of numerous neo-antigens and the consequent strong immune reaction against the tumor. In the MSI-high subgroup, the presence of BRAF^V600E^ does not change the prognosis. Moreover, in the metastatic phase, MSI-high tumors are the only responders to immune-checkpoint-inhibitor-based therapy, with a long-term stabilization of disease [4]. Unfortunately, the more frequently occurring MSI-low CRC has no specific therapies.

BRAF^V600E^ mutation is a strong, negative prognostic factor in stage III-IV, microsatellite-stable CRC. BRAF^V600E^ inhibitors, which are active against melanoma, have not shown any effect against CRC, as CRC can rapidly upregulate the wild-type BRAF allele, reactivating EGFR signaling. At the same time, BRAF^V600E^ metastatic CRC patients show a very poor outcome and do not benefit from Cetuximab therapy. In fact, BRAF^V600E^ is constitutively active and bypasses EGFR signaling, as is already observed for KRAS mutations. The merging of these strategies has led to better results. The combination of a BRAF^V600E^ inhibitor and Cetuximab in the BEACON phase III trial, when used as a second line of therapy for BRAF^V600E^ microsatellite-stable mCRC, increased the median overall survival from 5.9 to 9.3 months [4]. Despite this benefit, BRAF^V600E^ mCRC is far from being adequately treated, as compared to the responses of MSI-high patients.

While several intracellular signaling inhibitors have been tested to enforce a targeted approach against BRAF^V600E^ mCRC, some observations for organoid cultures point to an alternative strategy that could specifically target CMS1 CRC with high efficacy.

## 2. The Intriguing Observations from In Vitro Organoid Cultures

The paper by Xingru Li and colleagues [5] investigated the fascinating hypothesis that the adaptation of an organoid to in vitro culture could mirror its need for a specific niche, thus allowing new therapeutic targets to be identified.

The authors established 22 long-term CRC organoid cultures from 40 patients. The samples derived from different locations, stages and grades, and were cultured in the same defined medium. Organoids were considered as established cultures when they could be split five consecutive times and proliferate for one month in vitro. Established CRC organoids maintained a histology resembling the tumor of origin. The comparison of MSI, BRAF and KRAS status between organoids and matched primary tumors showed a good correspondence, with the only exception being one BRAF wild-type tumor generating a BRAF-mutated organoid, probably by way of the in vitro outgrowth of a scarcely represented population.

Established and non-established organoids were clustered according to clinical pathology and molecular subtypes. This classification immediately showed that most non-established cultures displayed the fingerprint of CMS1 tumors, enriched in MSI, BRAF-mutated and mucinous CRC. MSI and mucinous tumors perfectly corresponded to right colon localization, while BRAF-mutated tumors showed a single exception with left localization, among 11 positive, non-established samples. All MSI tumors were negative for beta-catenin activation (detected as nuclear staining in IHC), while BRAF-mutated tumors showed one positive exception. These data showed a low adaptation of CMS1 tumors to in vitro conditions and a possible link to beta-catenin signaling, mediating the subsistence of the stem cell niche.

In the following step, the authors used RNAseq to compare eight tumors with established organoids to eight tumors not established in vitro. The analysis also included six matched organoids. Most differentially regulated genes between the two groups of tumors were linked to stem cell fate and immune/inflammatory response. In organoid-forming tumors, *LGR6* (the LGR5 analog, marker of gut stem cells) and *TRIM71* (maintaining the growth of embryonic stem cells) were upregulated, while negative/alternative beta-catenin pathway regulators such as *DKK1* and *ROR2* were prevalent in tumors unable to adapt to in vitro conditions. Additionally, in tumors not permissive to organoid establishment, the TGFβ-pathway-related genes *BMPR1B* and *TGFBR3L* were upregulated. Curiously, while immune and inflammatory cytokines were prevalent in tumors not adapted to in vitro conditions, the IHC analysis of the tumor front in the two groups did not show substantial differences in the number of infiltrating cells (CD3, CD8, CD66b, CD68 and CD20 were evaluated). As highlighted by the authors, besides the number and the localization of infiltrating immune cells, it would be essential to define the cytokine milieu and the functional state of the different immune cell subsets found within the tumor. Furthermore, it would be useful to define whether the immune subsets can influence the fate of either healthy or cancer stem cells.

The comparison of tumors with the matched, established organoids showed a reduced signature in organoids, mainly linked to the absence of a tumor microenvironment, excluding fibroblast/leucocyte activity. tRNA aminoacylation, the first step in protein biosynthesis, was the only pathway prevailing in organoids, as compared to the tissue of origin.

Finally, in silico analysis was performed on the TGCA database. TGCA patients were subdivided on the basis of the gene signature obtained in the RNAseq analysis of the authors’ cohort, clustering tumors on the basis of their predicted ability to establish, or not, organoids in vitro. A non-statistically significant trend toward a worse prognosis was observed for patients bearing tumors able to adapt to in vitro culture. Though this effect could be linked to the lack of MSI CRC patients, usually showing a better prognosis, that were strongly enriched in the non-organoid-forming tumors cohort.

## 3. Can We Translate These Results to Clinics?

Despite a relatively small cohort, the authors found an almost absolute linkage between CMS1 CRC and the lack of adaptation to in vitro culture. In the title of their study, they correctly linked this effect to an inefficient support of the stem cell population. A previous study by Sato’s group [6] showed that MSI CRC is frequently dependent on Wnt3a and R-Spondin for in vitro establishment, while microsatellite-stable CRC is not. Wnt3a, R-Spondin and Noggin are necessary for the culture of normal colon mucosa to avoid a loss in LGR5+ stem cell population. While Wnt3a and R-Spondin are synergic agonists of the beta-catenin pathway, Noggin sequestrates autocrine bone morphogenic proteins (BMPs), reducing differentiating signals. These factors were not used by Li and colleagues, and probably made the difference in the non-established organoids group. RNAseq data sustain this hypothesis, showing not only the negative regulation of beta-catenin-related markers (confirmed by loss of beta-catenin nuclear staining in IHC), but also the influence of the BMP/TGFβ pathway. By chance, the small cohort studied by Li and colleagues was particularly enriched in CMS1 tumors. These samples, along with Sato’s data, confirm the rarity of CMS1 tumors showing independence from stem cell niche factors.

An interesting retrospective study by Antonia Strippoli and colleagues [7] could provide the rationale for the double targeting of EGFR signaling and the stem cell niche. The authors investigated the effectiveness of Cetuximab in treating mCRC with wild-type KRAS/BRAF signaling. In their cohort of 121 patients, high c-MYC was linked to worse prognosis. c-MYC is the main downstream transcript of beta-catenin signaling, and the authors found that c-MYC increased more frequently in the metastases treated with Cetuximab, as a mechanism of resistance against EGFR inhibition. While most non-CMS1 CRCs can rely on APC mutations to obtain a constant activation of the beta-catenin pathway, CMS1 tumors show extreme sensibility to the privation of the natural stem cell agonists, losing a fundamental mechanism of resistance. This targeting could be particularly relevant in aggressive BRAF^V600E^ mCRC without MSI, under double treatment with Cetuximab and BRAF inhibitor. In this setting, the targeting of the stem cell niche could make the difference between response or relapse. On the other side, the targeting of the stem cell niche in MSI-high tumors, treated by anti-checkpoint inhibitors, could turn stabilization to eradication.

While stem cell targeting remains a neglected approach at the clinical level, its specific development against CMS1 CRC could lead to promising results. This strategy could be optimized by the contemporary inhibition of beta-catenin signaling and the agonism of the BMP/TGFβ pathway, possibly driving tumor cells to terminal differentiation and senescence.

## References

[1] Fontana E., Eason K., Cervantes A., Salazar R., Sadanandam A. (2019). Context Matters—Consensus Molecular Subtypes of Colorectal Cancer as Biomarkers for Clinical Trials. Ann. Oncol..

[2] Marmorino F., Boccaccino A., Germani M.M., Falcone A., Cremolini C. (2020). Immune Checkpoint Inhibitors in PMMR Metastatic Colorectal Cancer: A Tough Challenge. Cancers.

[3] Mauri G., Bonazzina E., Amatu A., Tosi F., Bencardino K., Gori V., Massihnia D., Cipani T., Spina F., Ghezzi S. (2021). The Evolutionary Landscape of Treatment for BRAFV600E Mutant Metastatic Colorectal Cancer. Cancers.

[4] Koncina E., Haan S., Rauh S., Letellier E. (2020). Prognostic and Predictive Molecular Biomarkers for Colorectal Cancer: Updates and Challenges. Cancers.

[5] Li X., Larsson P., Ljuslinder I., Öhlund D., Myte R., Löfgren-Burström A., Zingmark C., Ling A., Edin S., Palmqvist R. (2020). Ex Vivo Organoid Cultures Reveal the Importance of the Tumor Microenvironment for Maintenance of Colorectal Cancer Stem Cells. Cancers.

[6] Fujii M., Shimokawa M., Date S., Takano A., Matano M., Nanki K., Ohta Y., Toshimitsu K., Nakazato Y., Kawasaki K. (2016). A Colorectal Tumor Organoid Library Demonstrates Progressive Loss of Niche Factor Requirements during Tumorigenesis. Cell Stem Cell.

[7] Strippoli A., Cocomazzi A., Basso M., Cenci T., Ricci R., Pierconti F., Cassano A., Fiorentino V., Barone C., Bria E. (2020). C-MYC Expression Is a Possible Keystone in the Colorectal Cancer Resistance to EGFR Inhibitors. Cancers.

